# Efficacy and safety of abaloparatide, denosumab, teriparatide, oral bisphosphonates, and intravenous bisphosphonates in the treatment of male osteoporosis: a systematic review and Bayesian network meta-analysis

**DOI:** 10.3389/fendo.2025.1558560

**Published:** 2025-04-16

**Authors:** Liangshi Chen, Bomei Ji, Cong Xia

**Affiliations:** ^1^ Orthopedics, The First People’s Hospital of Taicang, Taicang Affiliated Hospital of Soochow University, Suzhou, Jiangsu, China; ^2^ Anesthesiology and Surgery, The First People’s Hospital of Taicang, Taicang Affiliated Hospital of Soochow University, Suzhou, Jiangsu, China

**Keywords:** abaloparatide, denosumab, teriparatide, bisphosphonates, male osteoporosis

## Abstract

**Study Design:**

A Systematic Review and Bayesian Network Meta-Analysis

**Objective:**

To compare the efficacy and safety of abaloparatide (ABA), denosumab (DEN), teriparatide (TER), oral bisphosphonates (OBP), and intravenous bisphosphonates (IBP) in the treatment of male osteoporosis through a network meta-analysis.

**Summary of Background Data:**

Currently, a variety of medications are available for the treatment of male osteoporosis, including abaloparatide, denosumab, teriparatide, and bisphosphonates. These medications are widely applied in male osteoporosis, and existing randomized controlled trials (RCTs) provide strong evidence of their efficacy. However, there is a lack of sufficient systematic comparative studies to guide the choice between these treatments, particularly for specific male osteoporosis populations.

**Methods:**

This systematic review and network meta-analysis (NMA) were conducted strictly in accordance with the PRISMA (Preferred Reporting Items for Systematic Reviews and Meta-Analyses) guidelines and the relevant standards recommended by the Cochrane Collaboration. We performed pairwise meta-analysis using Stata 18.0 software to assess the magnitude of effect sizes and the consistency of findings across studies. For network meta-analysis (NMA), we used R version 4.3.1 along with the gemtc and BUGSnet packages to handle complex multi-treatment comparisons. Using these methods, we were able to comprehensively assess the relative efficacy and safety of different treatment options. All statistical analyses were conducted using Review Manager software (version 5.4), a widely used tool in medical research for meta-analysis, forest plot generation, and bias risk assessment.

**Results:**

Overall, clinical decisions should carefully balance drug efficacy and safety. Although TER performs best in reducing the occurrence of all adverse events, its efficacy in some BMD targets (such as total hip BMD) is relatively lower. In comparison, while OBP has a clear advantage in reducing severe adverse events, its efficacy in some BMD improvements (such as femoral neck BMD) is slightly less. Therefore, clinicians should consider the specific needs of the patient, the treatment goals, and the safety profile of the drug when selecting a medication, particularly for long-term use.

**Conclusion:**

The results indicate that abaloparatide and teriparatide are significantly superior to other drugs in improving lumbar spine and femoral neck BMD, while oral bisphosphonates is the most effective in improving total hip BMD. In terms of safety, teriparatide demonstrates the best performance in all adverse events, and oral bisphosphonates shows a clear advantage in reducing severe adverse events. Future treatment decisions should balance efficacy and safety, with clinical treatment tailored to the individual needs of the patient, including the site of bone loss and sensitivity to adverse events. Future research should explore combination therapies or multi-target strategies to optimize both efficacy and safety.

## 1 Introduction

Osteoporosis is a disease characterized by reduced bone mineral density and bone fragility, significantly increasing the risk of fractures (1, 2). Although osteoporosis primarily affects women, it also significantly impacts men, particularly in the elderly male population (3). According to epidemiological data, approximately 2 million men in the United States are affected by osteoporosis, and about 1 in 5 white men will experience an osteoporotic fracture during their lifetime (4). With the aging population, the prevalence of osteoporosis in men continues to rise annually (5, 6). The onset of male osteoporosis is often accompanied by a decrease in bone mineral density, especially in men aged 50 and above (7). Although the clinical presentation and treatment strategies for male osteoporosis have distinct features, its pathogenesis is similar to that of female osteoporosis, generally associated with excessive bone resorption or insufficient bone formation (8, 9). Primary osteoporosis refers to osteoporosis without an identifiable underlying disease, whereas secondary osteoporosis is related to other diseases or medication use (10). This study focuses on the treatment of primary male osteoporosis, particularly the treatment strategies in high-risk patients.

Currently, a variety of medications are available for the treatment of male osteoporosis, including abaloparatide, denosumab, teriparatide, and bisphosphonates (11). These medications each have distinct characteristics and have shown effective results in the treatment of male osteoporosis, though their indications, mechanisms, and effects may differ. Bisphosphonates (including both oral and intravenous formulations) remain one of the most widely used therapeutic options in clinical practice. Oral bisphosphonates include alendronate, risedronate, and ibandronate (12, 13), while intravenous bisphosphonates, such as zoledronic acid (14), effectively prevent bone loss by inhibiting bone resorption and demonstrate good efficacy and safety. Abaloparatide, denosumab, and teriparatide are relatively newer treatments used in clinical practice for male osteoporosis, promoting bone mineral density increases and reducing fracture risk through different mechanisms (15–18). This means that, in terms of pharmacological mechanisms, they belong to different classes of drugs overall. These medications are widely applied in male osteoporosis, and existing randomized controlled trials (RCTs) provide strong evidence of their efficacy. However, there is a lack of sufficient systematic comparative studies to guide the choice between these treatments, particularly for specific male osteoporosis populations.

Against this backdrop, this study aims to systematically evaluate and compare the efficacy and safety of abaloparatide (ABA), denosumab (DEN), teriparatide (TER), oral bisphosphonates (OBP), and intravenous bisphosphonates (IBP) in the treatment of male osteoporosis through a network meta-analysis. Specifically, we will examine their effects on bone mineral density improvement, fracture prevention, and adverse events. Through this analysis, we hope to provide an evidence-based, comprehensive treatment guideline to assist clinicians in making more precise decisions in the management of primary male osteoporosis.

## 2 Materials and methods

### 2.1 Search strategy

This systematic review and network meta-analysis (NMA) were conducted strictly in accordance with the PRISMA (Preferred Reporting Items for Systematic Reviews and Meta-Analyses) guidelines and the relevant standards recommended by the Cochrane Collaboration (19). These guidelines provide a detailed framework to ensure the transparency, scientific rigor, and consistency of systematic reviews and meta-analyses. The methodology for this review has been pre-registered on the PROSPERO platform with registration number CRD42024623547. For literature collection, we performed comprehensive electronic searches across multiple major databases, including Web of Science, PubMed, and the Cochrane Library, covering all relevant literature from the inception of these databases up to October 2024. To ensure completeness, the specific details and implementation steps of all search strategies are provided in the **Supplementary Data**. Additionally, to further minimize selection bias and ensure comprehensiveness, we manually searched and cross-checked the references of previously published pairwise meta-analyses.

### 2.2 Inclusion criteria and study design

This systematic review exclusively incorporates randomized controlled trials (RCTs) for network meta-analysis, while excluding non-original research formats, including case reports, review articles, letters to the editor, conference abstracts, opinion pieces, and study protocols. The study population consists of male patients with primary osteoporosis, and only studies related to primary osteoporosis were included, excluding those involving secondary osteoporosis (osteoporosis caused by gonadal dysfunction, corticosteroid use, cancer, or other systemic diseases).

The efficacy assessment was based on changes in bone mineral density (BMD) at the lumbar spine, femoral neck, and total hip, as well as the percentage change in BMD, to reflect the effects of different therapeutic drugs. Specifically, lumbar spine BMD is measured using dual-energy X-ray absorptiometry (DXA) or similar techniques to assess the mineral content in the lumbar spine, effectively evaluating bone density and strength in the lower spine region (20, 21). Total hip BMD measures the mineral content across the entire hip joint area, including the proximal femur and surrounding structures, providing insights into bone health and strength at the hip (22). It is worth noting that the effects of abaloparatide and teriparatide on total hip BMD may differ from those of bisphosphonate treatment, potentially due to artifact. This is because changes in patient positioning can influence the regional BMD assessed by DXA, leading to measurement variability. Femoral neck BMD measures the mineral content in the femoral neck, a high-risk area for hip fractures, and is significant for predicting fracture risk and bone health (23, 24).

Safety assessments were based on the occurrence of all adverse events (AEs) and serious adverse events (SAEs) (25, 26). All AEs include any negative health responses occurring during clinical trials or treatment, ranging from mild side effects (headache, nausea and local discomfort) to more significant health issues. For example, previous studies have reported upper gastrointestinal discomfort symptoms, such as nausea, vomiting, and epigastric pain, which may result from oral bisphosphonate therapy. In addition, there have been some reports of side effects such as dizziness and injection site reactions associated with teriparatide. Serious adverse events refer to health problems that may pose a life-threatening risk, including fatal events, severe disability, hospitalization, prolonged hospital stay, or even death.

### 2.3 Data extraction and quality assessment

Data collection was conducted independently by two researchers (XX and XX). During the data extraction process, the following key information was collected: first author, year of publication, study design type, country of origin, detailed information on interventions, sample size, and duration of follow-up. All collected data were organized and entered into an electronic database for subsequent analysis and processing.

For the patient outcome assessment, we primarily focused on the percentage change in bone mineral density (BMD) at the lumbar spine, femoral neck, and total hip to evaluate the efficacy of interventions. These BMD measures reflect critical aspects of bone health and are effective in assessing the impact of interventions on osteoporosis. Furthermore, to comprehensively assess the safety of interventions, we also recorded the occurrence of all adverse events (AEs) and serious adverse events (SAEs) during the treatment period. These data allowed us to evaluate the differences in efficacy and safety between different interventions. For studies with incomplete data, we strictly adhered to the guidance in Section 6.5.2 of the Cochrane Intervention Review Handbook and applied appropriate variance transformation or estimation methods to ensure data completeness and analytical accuracy (27). If the study reported mean differences and P-values, we calculated the standard error of the mean difference between groups using the formula in Section 6.5.2.3 and applied it in subsequent analyses. For studies where variance data were not provided, we assumed a conservative standard deviation of 30 for estimation (27). These steps were executed rigorously following scientific methodological standards to ensure the rigor and reliability of the data analysis.

Finally, to assess the quality of the included studies, we used the Cochrane Collaboration’s recommended bias risk assessment tool for randomized controlled trials (RCTs) (28–30). We performed a rigorous assessment of the risk of bias for each included study utilizing established methodological criteria, encompassing random sequence generation, allocation concealment, blinding of participants and personnel, blinding of outcome assessment, handling of incomplete outcome data, potential selective reporting, and other sources of bias. Based on the evaluation outcomes, each study was categorized as having a low risk, high risk, or unclear risk of bias to ensure transparency and methodological robustness.

It is important to note that in the event of any disputes or inconsistencies in data extraction or quality assessment, a third researcher (XX) intervened to resolve discrepancies through discussion and arbitration, ensuring the consistency and accuracy of the final data.

### 2.4 Statistical analysis

We performed pairwise meta-analysis using Stata 18.0 software to assess the magnitude of effect sizes and the consistency of findings across studies. For network meta-analysis (NMA), we used R version 4.3.1 along with the gemtc and BUGSnet packages to handle complex multi-treatment comparisons (31–33). Using these methods, we were able to comprehensively assess the relative efficacy and safety of different treatment options. All statistical analyses were conducted using Review Manager software (version 5.4), a widely used tool in medical research for meta-analysis, forest plot generation, and bias risk assessment.

In the data synthesis process, we selected appropriate statistical methods based on the type of data. For categorical variables, we used odds ratios (OR) to assess the relative risk between treatment and control groups, describing the difference in event rates between the two groups. The odds ratio is a commonly used statistic in clinical research that reflects the relative effect of an intervention in preventing a specific event. For continuous variables, we used mean difference (MD) to analyze the effect size between the treatment and control groups, revealing the impact of the intervention on specific physiological parameters such as bone mineral density (34). The mean difference effectively quantifies the changes before and after treatment and between groups, helping to evaluate the actual effects of the intervention.

## 3 Results

**Figure 1:**
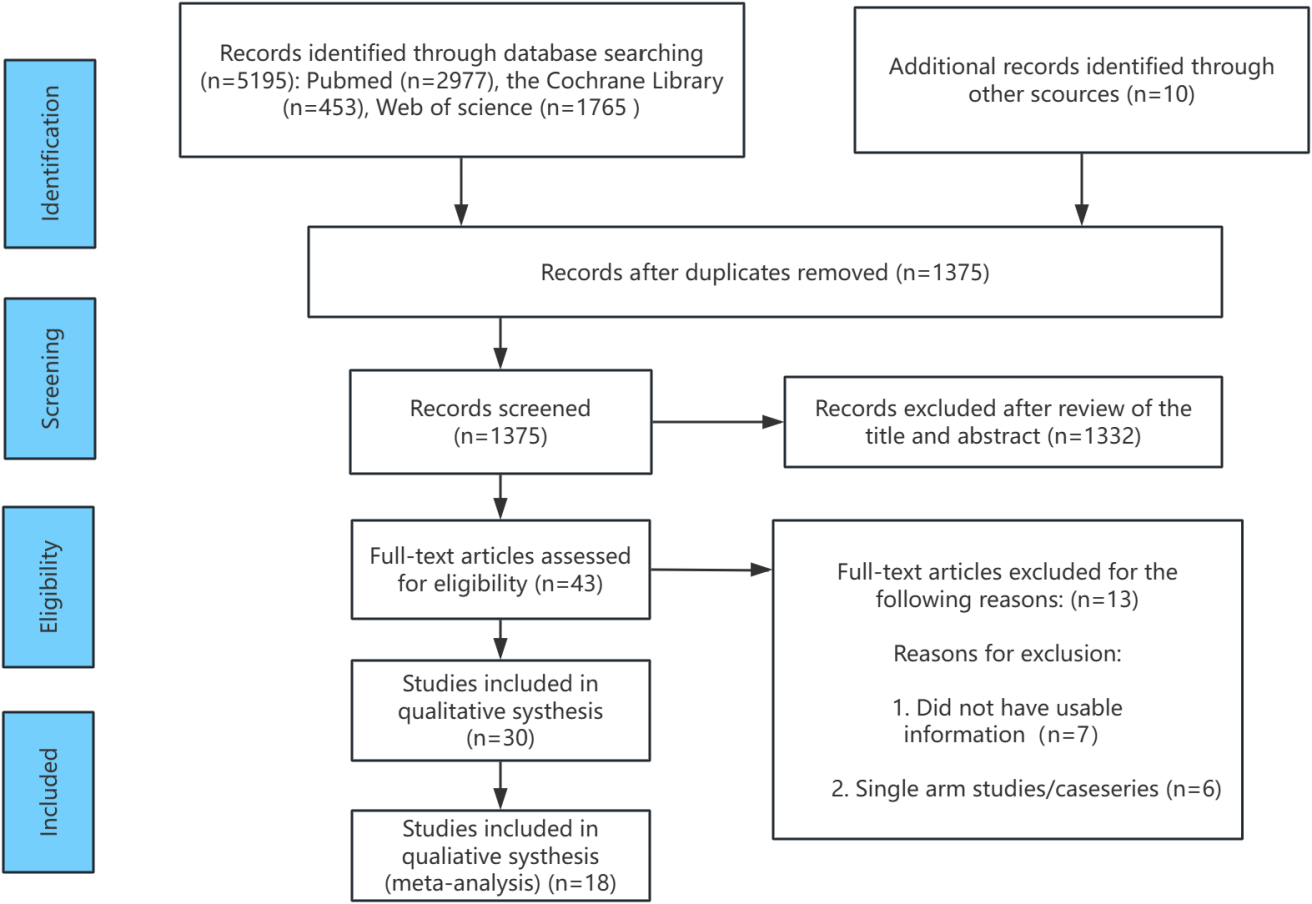
Flow chart of the selection process for relative studies in meta-analysis.

**Figure 1** presents the flowchart illustrating the screening process for the included relevant randomized controlled trials (RCTs). During the literature search, a total of 5195 records were identified. After removing duplicates (references that appeared in multiple databases), 1375 unique records remained for further evaluation. Following full-text review and the application of inclusion criteria, several studies with lower relevance to the research topic were excluded. For example, the studies by Ringe in 2006 (35) and 2009 (36) were conducted by the same research team at different time points. To avoid data duplication, the 2006 study was excluded. Studies such as Sambrook 2012 (37), Glüer 2013 (38), Reid 2000 (39), and Wallach 2000 (40) focused on osteoporosis in men treated with high-dose corticosteroids, which was not aligned with the focus of this study, and were therefore excluded. Additionally, studies by Smith 2003 (41) and Fizazi 2011 (42) investigated osteoporosis following estrogen deprivation therapy and testosterone deprivation therapy in prostate cancer patients, respectively. Since the clinical populations in these studies did not meet the inclusion criteria for this research, they were also excluded. Finally, Saag 2019 (43) and Nakamura 2014 (44) were excluded due to insufficient male patient data or unclear gender distinctions. After these exclusions, 18 RCTs involving 4392 participants were included in the analysis. These studies investigated five treatment regimens (oral bisphosphonates, intravenous bisphosphonates, abaloparatide, denosumab, and teriparatide), as well as placebo and alfacalcidol. These studies served as the foundation for the subsequent network meta-analysis.

**Figure 2:**
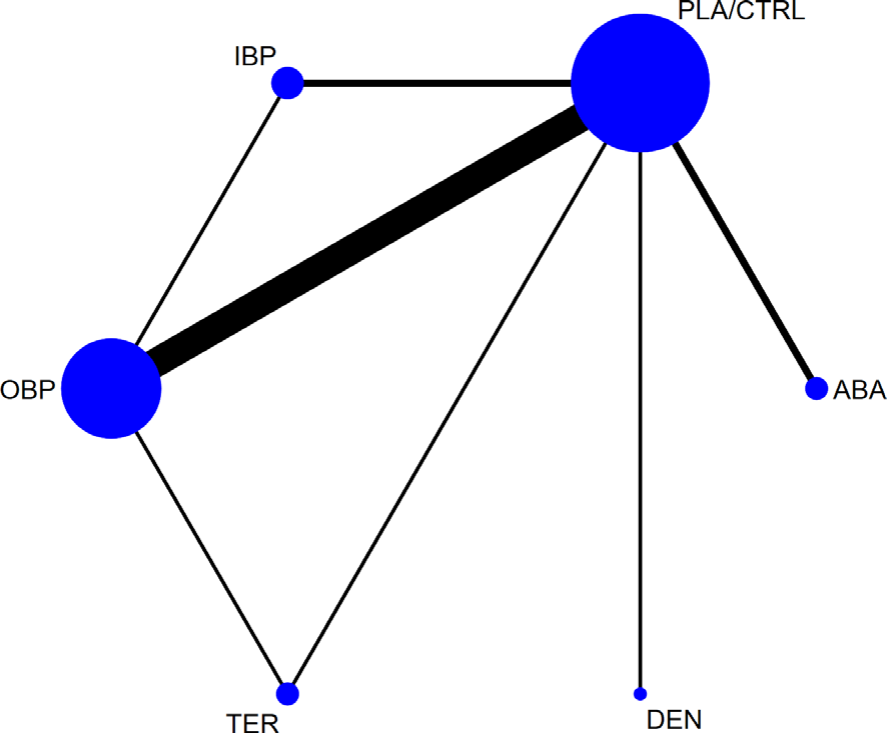
The network plot of all trials (ABA, abaloparatide; DEN, denosumab; TER, teriparatide; OBP, oral bisphosphonates; IBP, intravenous bisphosphonates; PLA/CTRL, placebo/control).

**Figure 2** illustrates the associated network diagram, where each osteoporosis treatment was compared with at least one placebo-controlled trial, and each treatment was directly compared with at least one active drug in the network. **Supplementary Table 1** shows the results of the statistical analysis, indicating good consistency across all outcomes, regardless of whether the consistency or inconsistency model was applied and the node-splitting method also did not detect significant inconsistency (P > 0.05). The primary outcomes, including lumbar spine, femoral neck, and total hip bone mineral density, had a follow-up period of 12 months, with fewer studies reporting longer or shorter follow-up durations. To ensure the reliability of the results, we used the follow-up data closest to 12 months for analysis. **Supplementary Figure 1** presents the forest plots for all outcomes, and **Supplementary Figure 2** displays the network plots for all outcomes. In the network plot, the size of the nodes represents the number of participants in each treatment group, while the thickness of the lines between nodes reflects the number of studies comparing those treatments. **Supplementary Figure 3** presents the funnel plots for bias analysis of all outcomes. Visual inspection of the funnel plots revealed no evidence of publication bias among the included studies.

### 3.1 Study characteristics and study quality

**Table 1** summarizes the 18 randomized controlled trials (RCTs) included in the network meta-analysis (NMA) and outlines their key characteristics (36, 45–61). These studies were published between 2000 and 2022, with male participant numbers ranging from 19 to 1199, and study durations spanning from 1 to 3 years. The majority of studies compared treatment regimens with a placebo and/or calcium and vitamin D, while some also included alfacalcidol as a control (PLA/CTRL). Direct comparisons among the four treatment regimens were limited.

**Table 1:** The main features of the articles included in the Network Meta-Analysis.

Author, year	Study design	Country	Treatment	Comparator	Background treatment	Age(Mean ± SD)	Group/Patient	Length of interv
Gonnelli 2003 (47)	RCT	Italy	Alendronate 10 mg, oral daily administration	No placebo	Ca (1000 mg) daily oral administration	57.1 ± 10.8	G1: 39 G2: 38	156 weeks (3-years)
Hwang 2010 (52)	RCT	China	Alendronate 70 mg, oral weekly administration	No placebo	Ca and Vit D supplement, daily oral administration	G1: 59.0 ± 3.9 G2: 55.3 ± 2.2	G1: 23 G2: 23	24 weeks
Miller 2004 (61)	RCT	USA, Multicentre	Alendronate 70 mg, weekly oral administration	Placebo	Ca as carbonate (500 mg) and Vit D (200 IU), daily oral administration	G1: 65.8 ± 10.7 G2: 66.7 ± 12.4	G1: 109 G2: 58	52 weeks (1 year)
Orwoll 2000 (46)	RCT	20 centers in the United States and 10 other countries	Alendronate 10 mg, daily oral administration	Placebo	Ca (500 mg) and Vit D (400 IU), daily oral administration	G1: 63 ± 13 G2: 63 ± 12	G1:146 G2: 95	104 weeks (2 years)
Ringe 2004 (48)	RCT	Germany	Alendronate 10 mg, oral daily administration	Alfacalcidol (1 μg daily)	Supplemental calcium (500 mg daily)	G1: 52.1 ± 10.9 G2: 53.3 ± 11.1	G1: 68 G2: 66	3 years
Ringe 2009 (36)	RCT	Germany	Risedronate 5 mg, oral daily administration	Daily alfacalcidol (1 microg)	Ca (1,000 mg) daily and Vit D (800 IU), daily oral administration	G1: 55.8 ± 10.5 G2: 58.0 ± 10.3	G1: 158 G2: 158	104 weeks (2 years)
Walker 2013 (49)	RCT	Columbia	Risedronate oral 35 mg, weekly oral administration	1: Teriparatide daily subcutaneous injection 20 µg 2: Combination of both	Ca (500 mg) and vit D (400 IU), daily oral administration	G1: 54.0 ± 2.0 G2: 51.6 ± 3.9 G3: 56.7 ± 4.9	G1: 10 G2: 9 G3: 10	78 weeks (18 months)
Boonen 2009 (57)	RCT	Multicenter study	Risedronate 35 mg, weekly oral administration	Placebo	Ca (1 g) and vit D (400–500 IU), twice daily	G1: 60 ± 11 G2: 62 ± 11	G1: 191 G2: 93	104 weeks (2 years)
Orwoll 2010 (55)	RCT	USA	Ibandronate 150 mg, oral monthly administration	Placebo	Ca (1 g) and vit D (400 IU) twice daily	G1: 63.9 ± 11.2 G2: 65.0± 10.6	G1: 85 G2: 47	52 weeks (1 year)
Orwoll 2003 (51)	RCT	37 centers in 11 countries	Teriparatide 20ug, subcutaneous daily injection	1: Teriparatide 40ug, subcutaneous daily injection 2: Placebo	Ca (1000 mg) and Vit D (400–1200 IU), daily oral administration	G1: 59 ± 13 G2: 58 ± 13 G3: 59 ± 13	G1: 151 G2: 139 G3: 147	52 weeks (1 year)
Qi 2021 (50)	RCT	China	Teriparatide 20 µg/day, daily subcutaneous injection	Alendronate 10 mg/day, oral daily administration	Ca and Vit D (dose not provided), daily oral administration	G1: 53.2 ± 4.1 G2: 54.7 ± 6.3	G1: 50 G2: 50	52 weeks (1 year)
Kaufman 2004 (60)	RCT	37 study sites in 11 countries	Teriparatide 20ug, subcutaneous daily injection	1: Teriparatide 40ug, subcutaneous daily injection 2: Placebo	Supplemental calcium (1,000 mg daily) and vitamin D (400–1,200 IU daily)	58.6 ± 12.9	G1: 22 G2: 20 G3: 37	18 months
Boonen 2011 (58)	RCT	International	Zoledronic Acid 5 mg, yearly intravenous injection	Placebo	Ca (1–1.5 g) and Vit D (400–800 IU), daily administration	G1:72.5 ± 10.3 G2: 72.6 ± 10.4	G1:248 G2: 260	104 weeks (2 years)
Boonen 2012 (56)	RCT	Multicenter study	Zoledronic acid 5 mg, yearly intravenous injection	Placebo	Ca (1 g) and Vit D (800–1000 IU), daily oral administration	G1: 66 ± 17.5 G2: 66 ± 17.5	G1: 588 G2: 611	104 weeks (2 years)
Orwoll 2010 (53)	RCT	North America, Australia	Zoledronic acid 5 mg, yearly intravenous injection	Alendronate 70 mg, oral daily administration	Ca (1 g) and Vit D (800–1000 IU), daily oral administration	G1: 64.5 ± 9.9 G2: 63.5 ± 11.0	G1: 154 G2: 148	104 weeks (2 years)
Czerwinski 2022 (54)	RCT	USA	Abaloparatide 80 µg, daily subcutaneous injection	Placebo	NR	G1: 68.5 ± 8.3 G2: 67.8 ± 8.5	G1: 149 G2: 79	52 weeks (1 year)
Matsumoto 2022 (45)	RCT	Japan	Abaloparatide 80 µg, daily subcutaneous self-injections	Placebo	Ca and Vit D supplement, daily oral administration	G1: 71.7 ± 4.4 G2: 70.8 ± 9.0	G1: 14 G2: 6	78 weeks (18 months)
Orwoll 2012 (59)	RCT	Multicentre study (North America and Europe)	Denosumab 60 mg, sub cutaneous injection every 6 months (q6m)	Placebo	Ca (≥ 1 g) and Vit D (≥ 800 IU), daily oral administration	G1: 64.9 ± 10.5 G2: 65.0 ± 9.1	G1: 121 G2: 121	52 weeks (1 year)

**Figure 3:**
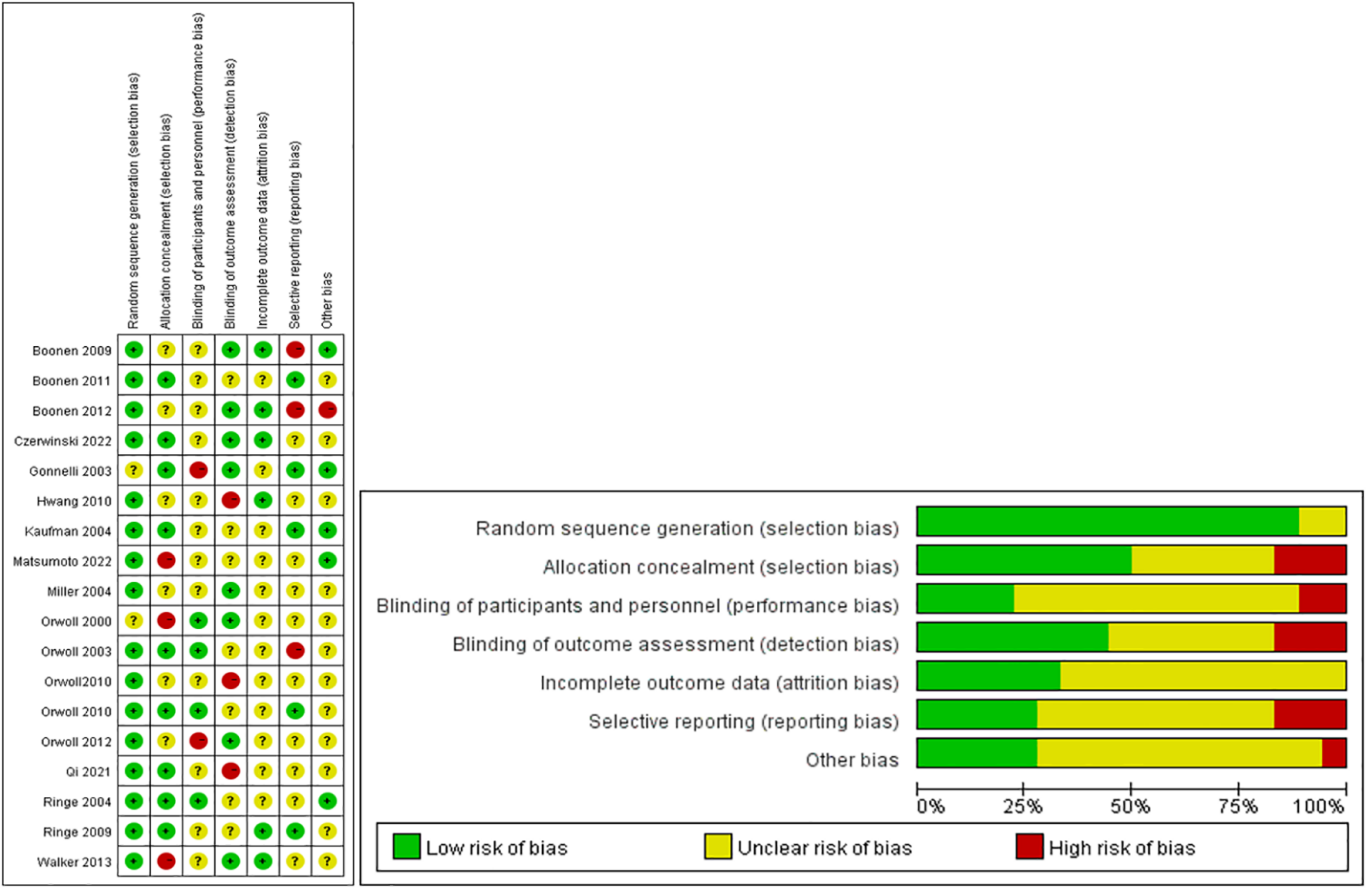
The network plot of all trials.Risk of bias summary for RCTs: Reviewers' judgments about each risk of bias item per included study.

**Figure 3** shows the risk of bias assessment for the included studies, conducted according to the Cochrane Collaboration guidelines. The assessment primarily focused on the random sequence generation, allocation concealment, and completeness of outcome reporting. Overall, most studies did not fully report the methods for random sequence generation and allocation concealment, particularly the details of the randomization process.

### 3.2 Lumbar spine BMD

**Figure 4:**
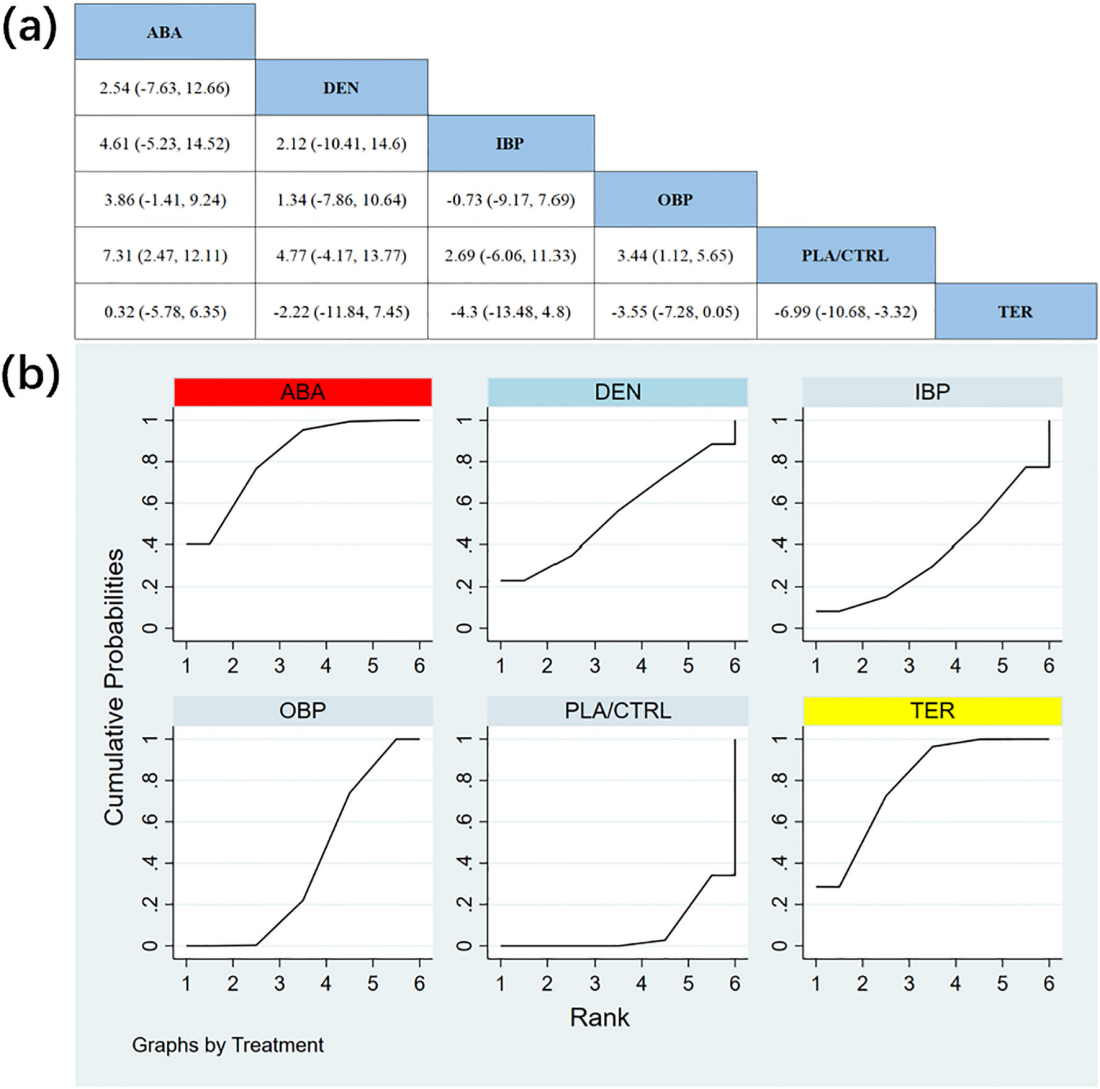
**(a)** The results of League table for Lumbar spine BMD. **(b)** Ranking the probability of Lumbar spine BMD percentage change.

In 12 randomized controlled trials (RCTs) with a total of 2,226 participants, we compared the effects of different treatments on lumbar spine bone mineral density (BMD). The results showed that the ABA and TER treatment groups significantly outperformed other drugs in improving lumbar spine BMD. **Figure 4a** shows the specific differences between the treatment groups. Further analysis revealed that, aside from ABA and TER, the differences between other treatment groups in lumbar spine BMD were not statistically significant.

To further quantify the effects of different treatments on lumbar spine BMD, we conducted a cumulative ranking curve (SUCRA) analysis. **Figure 4b** displays the results of lumbar spine BMD improvement, where red represents the highest rank, yellow represents second, and blue represents third. The ranking results revealed significant differences in the effectiveness of various drugs in improving lumbar spine BMD, with higher rankings indicating better treatment outcomes. Based on the SUCRA analysis, the probabilities of improving lumbar spine BMD, from highest to lowest, were as follows: ABA (SUCRA = 82.3%), TER (SUCRA = 79.5%), DEN (SUCRA = 55.2%), OBP (SUCRA = 39.3%), IBP (SUCRA = 36.4%), and PLA/CTRL (SUCRA = 7.4%).

### 3.3 Total hip BMD

**Figure 5:**
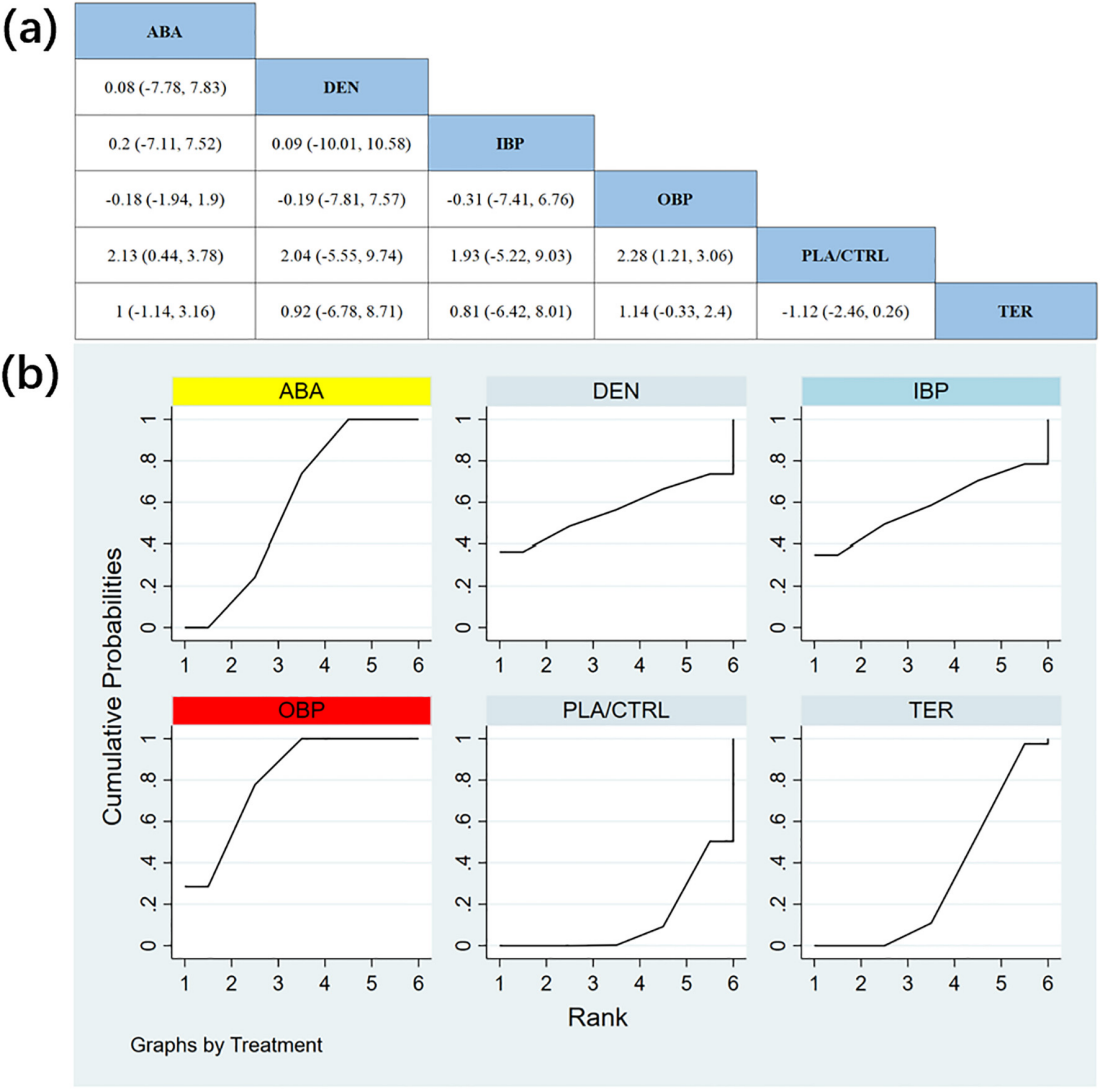
**(a)** The results of League table for Total hip BMD. **(b)** Ranking the probability of Total hip BMD percentage change.

In 11 RCTs with a total of 1,951 participants, we compared the effects of different treatments on total hip BMD. The results showed that the OBP treatment group significantly outperformed other groups in improving total hip BMD. **Figure 5a** shows the specific differences between the treatment groups. Further analysis revealed that, aside from OBP, the differences between other treatment groups did not reach statistical significance.

To further quantify the effects of different treatments on total hip BMD, we applied the cumulative ranking curve (SUCRA) method. **Figure 5b** displays the results of total hip BMD improvement. According to the SUCRA analysis, the probabilities of improving total hip BMD, from highest to lowest, were as follows: OBP (SUCRA = 81.3%), ABA (SUCRA = 59.6%), IBP (SUCRA = 58.4%), DEN (SUCRA = 56.3%), TER (SUCRA = 32.5%), and PLA/CTRL (SUCRA = 12.0%).

### 3.4 Femoral neck BMD

**Figure 6:**
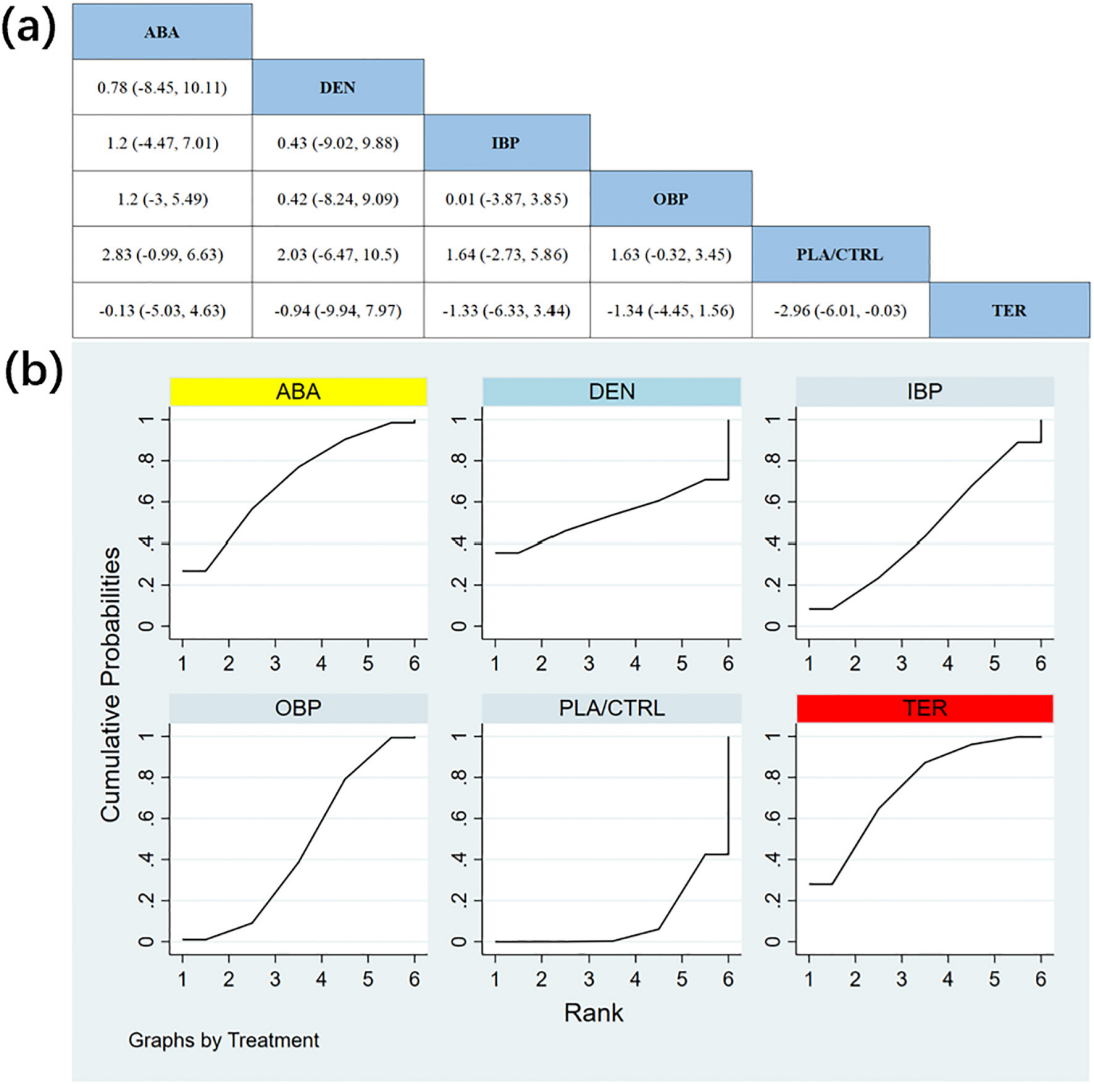
**(a)** The results of League table for Femoral neck BMD. **(b)** Ranking the probability of Femoral neck BMD percentage change.

In 12 RCTs with a total of 2,235 participants, we compared the effects of different treatments on femoral neck BMD. The results showed that the TER and ABA treatment groups significantly outperformed other treatment groups in improving femoral neck BMD. **Figure 6a** shows the specific differences between the treatment groups.

To further quantify the effects of different treatments on femoral neck BMD, we used the cumulative ranking curve (SUCRA) method to rank the treatments. **Figure 6b** displays the results of femoral neck BMD improvement. Based on the SUCRA analysis, the probabilities of improving femoral neck BMD, from highest to lowest, were as follows: TER (SUCRA = 75.2%), ABA (SUCRA = 69.8%), DEN (SUCRA = 53.3%), IBP (SUCRA = 46.4%), OBP (SUCRA = 45.5%), and PLA/CTRL (SUCRA = 9.8%).

### 3.5 All adverse events

**Figure 7:**
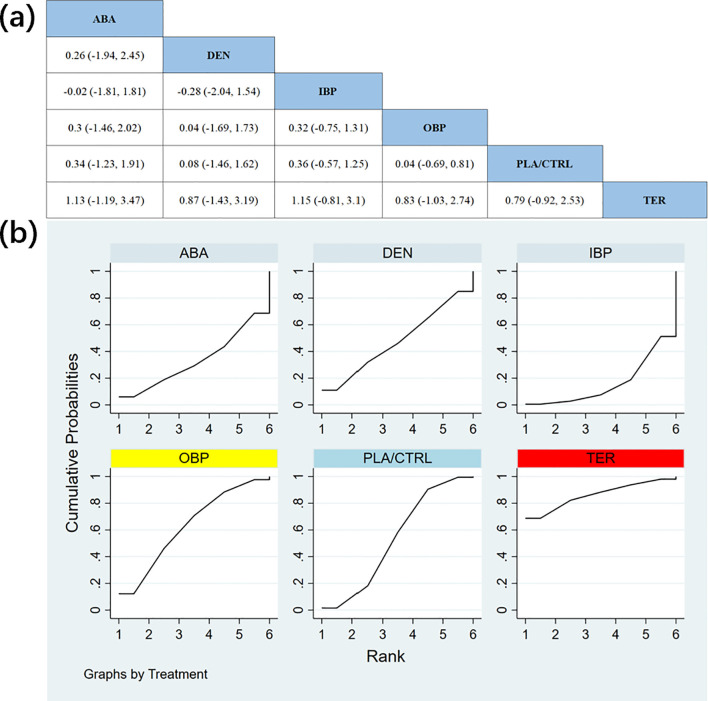
**(a)** The results of League table for all adverse events. **(b)** Ranking the probability of all adverse events.

In 10 RCTs with a total of 3,437 participants, we compared all adverse events (AEs) across different treatments. The results showed that the TER treatment group demonstrated significantly better safety than other treatment groups. **Figure 7a** shows the specific differences between the treatment groups.

To further quantify the safety of different treatments regarding all adverse events, we used the cumulative ranking curve (SUCRA) method. **Figure 7b** displays the results of all adverse events. According to the SUCRA analysis, the safety ranking probabilities for all adverse events, from highest to lowest, were as follows: TER (SUCRA = 86.2%), OBP (SUCRA = 63.0%), PLA/CTRL (SUCRA = 53.6%), DEN (SUCRA = 47.8%), ABA (SUCRA = 33.2%), and IBP (SUCRA = 16.2%).

### 3.6 Serious adverse events

**Figure 8:**
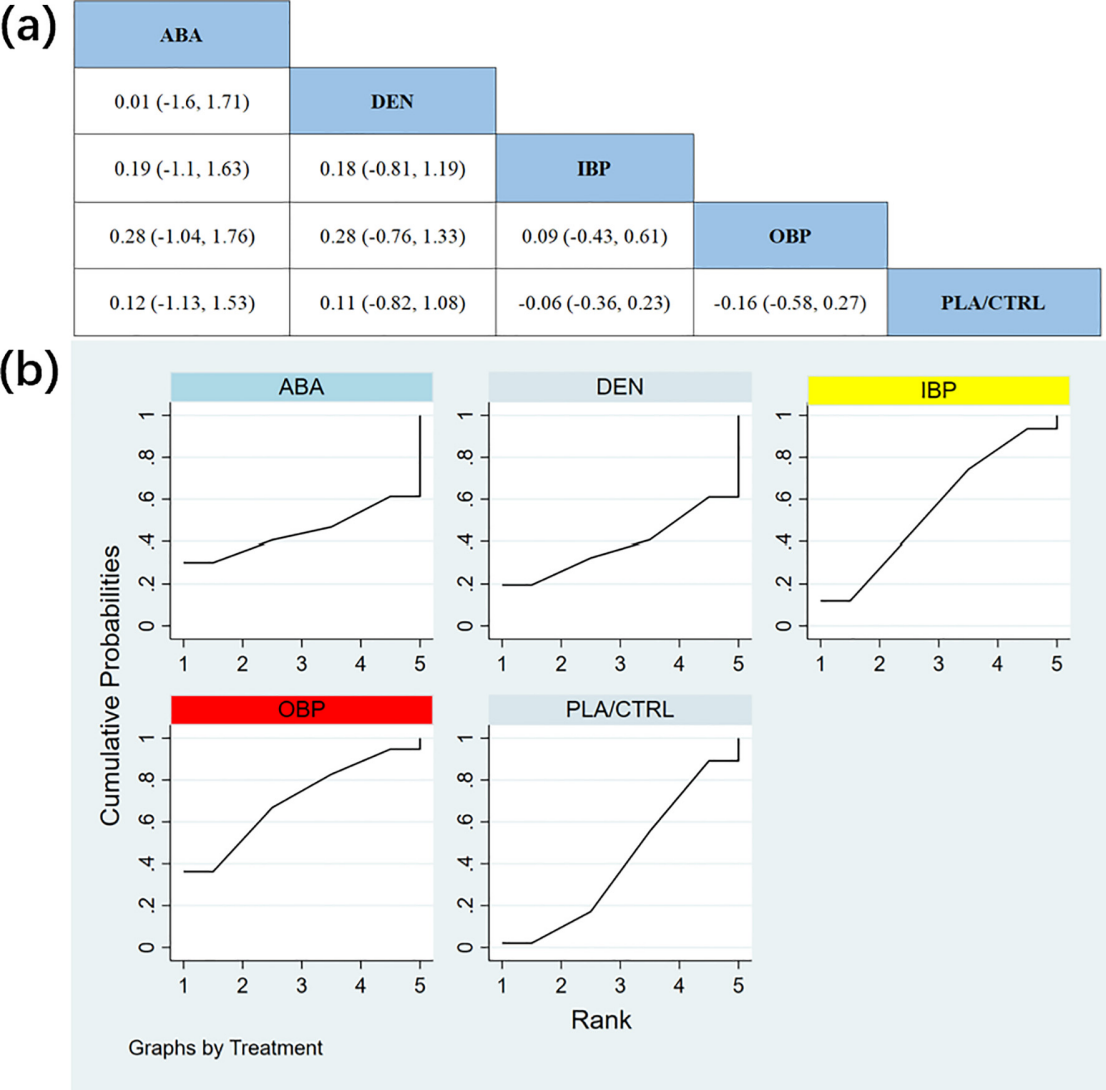
**(a)** The results of League table for serious adverse events. **(b)** Ranking the probability of serious adverse events.

In 8 RCTs with a total of 3,000 participants, we compared serious adverse events (SAEs) across different treatments (TER was excluded due to insufficient data). The results showed that the OBP treatment group demonstrated significantly better safety in terms of SAEs compared to other treatment groups. **Figure 8a** shows the specific differences between the treatment groups.

To further quantify the safety of different treatments regarding serious adverse events, we used the cumulative ranking curve (SUCRA) method. **Figure 8b** displays the results of serious adverse events. According to the SUCRA analysis, the safety ranking probabilities for serious adverse events, from highest to lowest, were as follows: OBP (SUCRA = 70.2%), IBP (SUCRA = 55.7%), ABA (SUCRA = 44.7%), PLA/CTRL (SUCRA = 41.0%), and DEN (SUCRA = 38.4%).

## 4 Discussion

This systematic review and network meta-analysis (NMA) provides a comprehensive evaluation of the efficacy and safety of several drugs (ABA, DEN, TER, OBP and IBP) in the treatment of primary osteoporosis in men.

In terms of efficacy, ABA and TER significantly outperform other drugs in improving lumbar spine and femoral neck BMD, while OBP shows the best performance in improving total hip BMD. These results suggest that different drugs or treatments may have distinct mechanisms of action when targeting BMD in different skeletal sites. For example, although ABA and TER show outstanding effects in improving lumbar spine and femoral neck BMD, their impact on total hip BMD is relatively weaker. In contrast, OBP has a significant effect on improving total hip BMD, indicating the selective effects of different drugs on BMD at different sites. This implies that, in the treatment of osteoporosis, the choice of drug should be based on the patient’s specific condition and the sites of BMD change, allowing for more personalized treatment. A tailored treatment strategy could enhance treatment outcomes and clinical satisfaction. It is particularly noteworthy that in the comparison of oral and intravenous bisphosphonates, oral bisphosphonates demonstrate a statistically significant advantage in improving total hip BMD. However, no significant differences in efficacy were observed between oral and intravenous bisphosphonates in terms of lumbar spine and femoral neck BMD.

Regarding safety, TER shows a distinct advantage in the incidence of all adverse events, suggesting that TER not only improves BMD effectively but also has better safety during treatment. This implies that TER could be an ideal choice for long-term therapy, especially for patients requiring prolonged drug treatment. The lower incidence of adverse events significantly improves patient adherence to treatment, thereby enhancing their quality of life. However, while TER excels in reducing all adverse events, OBP shows the most significant advantage in reducing the occurrence of severe adverse events. This suggests that OBP may offer higher safety in preventing serious side effects, particularly for high-risk patients (such as elderly individuals or those with multiple comorbidities), for whom OBP could be a safer treatment option. Additionally, oral bisphosphonates have a safety advantage over intravenous bisphosphonates, especially in reducing the occurrence of severe adverse events.

Overall, clinical decisions should carefully balance drug efficacy and safety. Although TER performs best in reducing the occurrence of all adverse events, its efficacy in some BMD targets (such as total hip BMD) is relatively lower. In comparison, while OBP has a clear advantage in reducing severe adverse events, its efficacy in some BMD improvements (such as femoral neck BMD) is slightly less. Therefore, clinicians should consider the specific needs of the patient, the treatment goals, and the safety profile of the drug when selecting a medication, particularly for long-term use.

Finally, in the comparison of oral versus intravenous bisphosphonates, Louise S. Conwell et al. (62) evaluated the efficacy of bisphosphonates in treating osteoporosis in cystic fibrosis patients through a meta-analysis and found that intravenous bisphosphonates might lead to severe bone pain and flu-like symptoms. This result is consistent with the findings of our network meta-analysis and further supports the greater efficacy and safety of oral bisphosphonates compared to intravenous bisphosphonates in the treatment of primary osteoporosis in men.

In recent years, the incidence of osteoporosis in men has gradually increased, prompting a rise in related clinical research, particularly systematic reviews and network meta-analyses (NMA). Managing osteoporosis in men still faces many challenges, and while existing studies focus on different aspects of drug interventions, fewer studies have addressed the differences in treatment effects between intravenous and oral bisphosphonates. Thus, this study employed a network meta-analysis to comprehensively assess and rank the efficacy and safety of five commonly used drugs in the treatment of male osteoporosis, providing more clinically relevant evidence. This network meta-analysis has several strengths (1): It included 18 studies with a total of 4392 participants, all of which were randomized controlled trials (RCTs), ensuring the reliability and scientific rigor of the data (2); It made a more detailed classification of drugs, dividing bisphosphonates into intravenous and oral categories, further revealing the treatment differences between different formulations and offering more directions for future research (3); The statistical results showed good consistency, reflecting the stability of the methodology, and through indirect comparisons of different treatment regimens, it provided a comprehensive evaluation of drug management in male osteoporosis. However, this study also has some limitations: (1) Some treatment drugs lack direct head-to-head comparisons, which may affect the rigor and reliability of the results and conclusions; (2) Data on severe adverse events for certain drugs (TER) were limited and not included in the analysis, which may impact the comprehensive assessment of drug safety; (3) The studies included in this research spanned a long period (2000–2022), which may lead to differences in study design, patient characteristics, and data collection methods, potentially affecting the quality of the results; (4) Due to the large variety of oral bisphosphonates and the scattered nature of the available data, subgroup analyses were not performed.

Ultimately, to further optimize the treatment outcomes and reduce drug side effects in male osteoporosis, there is a pressing need for more high-quality RCTs. These studies can assist physicians in developing more personalized treatment plans based on the patient’s specific condition, BMD assessment results, and potential risks, thereby achieving the goals of precision medicine and improving the efficacy and safety of clinical treatments.

## 5 Conclusion

The results indicate that abaloparatide and teriparatide are significantly superior to other drugs in improving lumbar spine and femoral neck BMD, while oral bisphosphonates is the most effective in improving total hip BMD. In terms of safety, teriparatide demonstrates the best performance in all adverse events, and oral bisphosphonates shows a clear advantage in reducing severe adverse events. Future treatment decisions should balance efficacy and safety, with clinical treatment tailored to the individual needs of the patient, including the site of bone loss and sensitivity to adverse events. Future research should explore combination therapies or multi-target strategies to optimize both efficacy and safety.

## Data Availability

The original contributions presented in the study are included in the article/**Supplementary Material**. Further inquiries can be directed to the corresponding author.

## Supplementary material

Supplementary Table 1 The results of compareing the deviance information criteria (DIC) between the consistency and inconsistency models.
